# The Foreign Journals: Pathology, Practical Medicine, and Therapeutics

**Published:** 1842-10

**Authors:** 


### PATHOLOGY, PRACTICAL MEDICINE, AND THERAPEUTICS.

*Case of Laceration of the Pulmonary Artery.* By Dr. HELMBRECHT.

A pontooner (pontonier), twenty-one years and half old, who had enjoyed good health, with the exception of slight dyspnœa during the three months that he had been in the army, and was quite equal to his laborious duties, was ordered to accompany his regiment on their march from Glogau to Mayence. On the route slight dyspnœa was his only ailment; and on December 5th having been engaged in carrying wood, he went to bed in good health. In the night he was roused by violent pain at the right side of the sternum with great dyspnœa, which subsided in the course of a few minutes, leaving him pale and cold, with a face expressing great anxiety and bathed in cold perspiration. After a short time nothing remained of his former condition but a sense of uneasiness in the precordial region, with very slight palpitation and remarkable feebleness of voice. He was bled, a mustard poultice was applied to the chest, and a mixture was given, containing sulphuric æther. During the afternoon of the following day he seemed well, but at eleven p. m. complained of sense of exhaustion and asked for drink. The attendant noticed his face become pale, and finding his feet cold, fetched the doctor. His pulse was then imperceptible, and the surface of the body cold, and in a few minutes the patient was dead.

On examining the body a large quantity of coagulated blood was found in the pericardium. The heart was healthy, the wall of the left ventricle rather thick. Minute calcareous concretions existed on the outer side of the pulmonary artery close to its origin, and extending towards the right ventricle. The inner coat of the artery was separated from the elastic coat for the space of three inches from the point where it joins the right ventricle, and torn into shreds which projected into and narrowed its cavity. The whole lining membrane of the artery was coated with a thin layer of fibrine. At its origin from the ventricle, exactly in the situation where the concretions terminated, was a hole of the size of a fourpenny-piece, through which the blood had escaped into the pericardium. The neighbourhood of this opening was of a deep red colour. The lungs were healthy and bloodless.

*Casper's Wochenschrift. March* 5, 1842.

---

*On Softening of the Brain.* By Dr. BUDGE.

THE writer questions the inflammatory origin of the colourless softening of the brain, though he admits it in the more frequent case in which there is a manifest rose tint of the cerebral substance. In confirmation of this opinion he appeals to the investigations of Gluge, who, while he discovered in both kinds of softening of the brain a partial destruction of the nervous fibrillæ, could not detect any exudation corpuscles in the capillaries of the brain in the white softening, though they were very evident in the red softening.

In the red softening the circulation becomes suspended in some of the capillaries of the brain. The blood-corpuscles accumulate within the vessels while serum transudes through their walls and renders the nervous substance soft and pultaceous. A change, too, usually takes place in the blood-globules themselves, by which their colouring matter becomes dissolved in the serum, and imparts to it a red hue. This must result from some alteration in the serum; probably from a diminution in the quantity of chloride of soda, which has a principal share in producing the insolubility of the colouring matter. The hæmatin of the blood-corpuscles would thus become dissolved in the liquor sanguinis, and occasion the red serum, while the altered blood-globules, probably from those larger compound bodies to which Gluge gave the name of compound exudation corpuscles, and which Weber termed round, cohering granules. It is, indeed, probable that the inflammatory process gives rise to a decomposition of the chloride of soda, the hydrochloric acid being eliminated from the blood by the stomach and giving rise to the impairment of the digestive functions which attends the inflammation of important organs, while the soda might produce a change in the albumen by which it is rendered more analogous to fibrin; a change for the occurrence of which many facts might be adduced.

The symptoms attending softening of the brain may be referred to two classes. 1st, Those which result from the nature of the disease—that is, from the partial destruction of the fibres of the brain. These may again be subdivided into two classes, according to whether they are symptoms of *paralysis* or are produced by *antagonism.* 2d, Symptoms which depend on the seat of the disease; and, unfortunately, in the present state of our knowledge these cannot be stated with certainty. In conclusion, a case is related in which the symptoms during life were referred to disease of the cerebellum, and in which that organ was found after death in a state of general softening.

*Organ für d. ges. Med. Bonn.* Bd. i. Hft. 3.

---

### *Monomania Ophthalmica.* By Dr. Winternitz.

A gentleman forty-nine years old, was seized with gout in 1837, which he himself was inclined to attribute to cold applications made to an accidental swelling of the head. But the simultaneous appearance of an obstinate paronychia on his right middle finger, together with the decline of his strength, earthy complexion, and emaciation, left no doubt as to the constitutional nature of the affection. Late in the autumn of 1840 he made a journey to north Germany in an open carriage, by which exposure he suffered from rheumatism. The abdominal organs became disordered, and about the same time he suffered morally from anxiety. All these concurrent circumstances had the effect of developing the hypochondriac diathesis in the patient. The disease which forms the subject of this article began in July, 1841. Dr. Winternitz found him in a state of the greatest uneasiness, complaining of a confusion of head. During the day and while in the act of walking he had frequent attacks of faintness, followed by general perspiration. He was sensible of a sudden spasmodic constriction of the right eye-ball, as if it were confined and pressed in the socket, accompanied by an actual twitching upward and downward of the upper eyelid. Objects appeared as if surrounded by a gauzy halo: the patient was unable to read, in consequence of the letters appearing to swim before him. Writing was less difficult, although he was continually apt to carry the pen beyond the paper. The eye itself, beyond a slightly diminished activity of the lachrymal gland, betrayed nothing morbid.

The author's opinion that the affection was of an hypochondriac or psychical origin was founded on the following circumstances. There were no objective symptoms; the patient's whole attention was engrossed by his disease, which he was disposed to consider by turns of a congestive, nervous, arthritico-metastatic, &c., origin; he sought eagerly the society and conversation of oculists; disconsolately anticipated the total loss of vision; the patient had, moreover, at a former period suffered from hypochondria.

The therapeutic indication was to withdraw the patient, as it were, from himself, and to interrupt his habit of constantly thinking of his disease. This was effected chiefly by means of a jaunt to Paris. Here the patient was advised to employ tepid camomile-flower baths, with cold water douches along the spine. Vertigo became of rarer occurrence; he gradually regained facility in reading and writing; and after three weeks' residence in Paris felt a strong desire to revisit his family, whom he reached in perfect health.

*Oesterreich. Medicinische Wochenschrift. Februar* 12, 1842.

---

*Rupture of the Trachea.* By Dr. Bredschneider, of Fischhausen.

The patient was a child one year and three quarters old, who was suffering from bronchitis with some signs of hydrocephalus, among which was that of incessantly and violently tossing its head about. On the 5th day of the disease a considerable tumour formed suddenly over the neck and chest. It began below the cricoid cartilage, reached upwards to the right ear, and extended downwards over both sides of the chest. A small incision was made into it, air escaped with a hissing noise, and, by the aid of pressure, the tumour collapsed. Two days after this the child died, and a rent was found in the trachea, half an inch long, on the right side, below the first cartilaginous ring. Was this produced by the violent movement of the head, or by coughing?

*Casper's Wochenschrift. Juli* 9, 1842.

---

*Tic-Douloureux from a Tumour beneath the Cerebellum.*
By Professor G. Barilli.

This paper was read at the meeting of the Academia delle Scienze, of Bologna, on the 12th of March. It relates to a lady, forty-two years old, of acute sensibility, in whom, after some inflammatory affection of the right side, symptoms of neuralgia in the right side of the face commenced. They gradually increased in severity, and, with two remissions—one of three the other of five months—they lasted till the time of her death, which occurred in the midst of the most severe sufferings, two years from the beginning of the neuralgia. The most striking characters of the affection were paroxysms of pain, preceded by a kind of aura and formication from the right temple to the gum of the same side, and violent tremulous spasm of the right temporal and buccal muscles. The sense of taste was also, during the paroxysms, lost. After death the blood-vessels of the brain and its membranes were found turgid. There was a tumour of a spheroidal form, two thirds of an inch in diameter, firmly adhering to the posterior-superior lamina of the tentorium, close by the sella turcica, and with its upper part lying in an indentation on the anterior margin of the right crus cerebelli. It was composed of a pulpy substance, deposited between numerous membranous partitions, and had a small plate of osseous matter at its base.

The author remarks, that the morbid appearances illustrated clearly the nature of the symptoms. One of the nerves affected was the motor division of the fifth, which Palletta has shown to arise by two filaments from the anterior margin of the crus cerebelli: on this the tumour pressed so as to push it from its usual direction. The sensitive branches of the fifth also were affected in the same proportion as they were subjected to the pressure of the tumour.

*Bulletino delle Scienze Mediche. Marzo*, 1842.

---

*Case of Peritonitis, in which the effused Fluid escaped through the Umbilicus.*
By Dr. Beonhardi, of Eilenburg.

A healthy girl, aged 5 years, was attacked on January 4, 1841, with symptoms of catarrhal fever. On January 6, the disease put on a more serious aspect, and signs of peritonitis came on. Active treatment, by local depletion, the administration of calomel, and mercurial inunction, was employed, and followed by marked improvement, but not by complete recovery. Some degree of fever still remained, the abdomen continued painful and grew distended, while percussion proved that this distension was not owing to the presence of air in the intestines but was produced by some liquid body.

No remedial measures could be adopted, the child obstinately refusing all medicine, and the condition of the patient became daily worse and worse. On January 22, the umbilicus became prominent, semitransparent, and red; and on the following day the tumour had increased to the size of a hen's egg, and fluid was distinctly perceptible through the attenuated skin. On the 25th the swelling burst and a quart and a half of fluid, partly purulent, escaped in a stream of the size of a goose quill, and an equal quantity drained away by degrees on the following day. The abdomen still continued tender, and the febrile symptoms were unrelieved, but the child now submitted to take small doses of calomel, which were continued till the 12th of February. A great improvement in the condition of the patient now took place, but the opening in the abdomen continued unclosed, and still gave exit to fluid though not in large quantity. Dr. B. waited till September, when he applied a graduated compress and elastic bandage. In fourteen days the opening closed, but the child still wears an umbilical hernia bandage, the destruction of the cellular tissue, by which the umbilicus was closed having permitted a protrusion of the intestines to take place.

*Medicinische Zeitung. March* 9, 1842.

---

*Apparent Death by Lightning.* By Dr. HARTMANN, of Neu Ruppin.

THREE persons were at the same instant struck by lightning. In one the symptoms were severe and remarkable. He was a healthy man of twenty-six. When the author saw him, an hour and half after the stroke, he lay completely unconscious, as if in apoplexy. His pulse was less than 60, full and hard; his respiration snoring; his pupils dilated and insensible. There were frequent twitchings of the arms and hands; the thumbs were flexed and immoveable; and the jaws firmly clenched. Soon after the author arrived, severe clonic spasms came on, so that four men could scarcely hold the patient in his bed; and his body was drawn to the left side. As soon as these had relaxed he was bled to 16 oz., cold was applied to the head, a blister to the nape, and mustard poultices to the legs. Stimulating enemeta and opium were also administered; and in the course of twenty-four hours the patient's consciousness slowly returned, and he was soon completely recovered. The only external injury discernible was a red streak, as broad as a finger, which extended from the left temple over the neck and the sternum, to the precordial region, and which disappeared completely in a few days.

*Medicinische Zeitung. Juni* 15, 1842.

---

*On a very simple means of arresting Epistaxis.* By Dr. NEGRIER, of Angers.

THIS consists in nothing more than closing with the opposite hand the nostril from which the blood flows, while the arm of the same side is raised perpendicularly above the head. In every instance in which he has had recourse to this means during the past three years M. Négrier has always found that it suspended the hemorrhage: a fact of which he offers the following explanation.

When a person stands in the ordinary posture, with his arms hanging down, the force needed to propel the blood through his upper extremities is about half that which would be required if his arms were raised perpendicularly above his head. But since the force which sends the blood through the carotid arteries is the same as that which causes it to circulate through the brachial arteries, and there is nothing in the mere position of the arms above the head to stimulate the heart to increased action, it is evident that a less vigorous circulation through the carotids must result from the increased force required to carry on the circulation through the upper extremities.

*Archives Générales de Médecine. June,* 1842.

*On the difference between true Typhus and Typhus Abdominalis, and on the latter affection in Children.* By Professor Seidlitz, of St. Petersburg.

Great confusion has arisen from the application of the word typhus to diseases essentially distinct and differing from each other, as typhus abdominalis and true typhus—typhus sanguineus.

The difference between the two diseases may be summed up somewhat in the following manner:

Typhus sanguineus is characterized by a short stage of premonitory symptoms; the rapid development and increase of the disease, marked by some striking though temporary symptom, as shivering; pain in the head; vomiting; extraordinary loss of strength. There is violent disturbance of the circulatory system; the blood is black, altered in character; and a great tendency exists to congestion of the brain, liver, and intestines, constituting hypostatic inflammations or apoplexies of those organs. The skin becomes burning hot, and an eruption of an erysipelatous character appears in large patches over the whole body. The urine is in moderate quantity, turbid, and loaded with crystalline deposits. The disease is contagious; it observes in its course a distinct septemeral type; and critical evacuations precede its termination.

Typhus abdominalis is characterized by long previous indisposition, gradual development and slow progress of the disease, with especial disturbance of the digestive powers. Excitement of the circulatory system does not come on till afterwards; and the blood, which is almost normal in character, does not become concentrated about the parenchymatous organs, but a tendency exists to serous effusion. Cerebral affections occur at a later period, and are evidently secondary. There is no disagreeable heat of skin, but rather a diminution of temperature; petechiæ are scattered about the lower part of the abdomen; and miliary eruptions appear towards the end of the disease. The urine is straw-coloured, and, at the end of the attack, deposits a sediment which resembles spiders' web. The disease is not contagious. Its course is very uncertain: it exceeds four weeks, and sometimes continues for six months. Sleep and a constipated state of the bowels announce its termination.

At different ages the symptoms of abdominal typhus vary considerably. One form which has usually not been referred to this head is that nervous fever which occurs in children between their sixth and their eighth year. Its precursory symptoms, which consist in irregular appetite, a variable state of the bowels, occasional attacks of fever, marked emaciation, dryness and harshness of the skin, unquiet sleep, and pettishness in the child's manner, often continue for several weeks and then disappear completely during the heats of summer, or, if they made their appearance in autumn, on the cold of winter setting in. Sometimes with the return of spring or autumn these symptoms recur, though less severely; and each year they reappear with diminished severity, till at last they cease entirely. At other times, however, under the influence of errors in diet or of moral causes—as the wearisomeness of a child's tasks at a boarding-school—the appetite is entirely lost, the evacuations become liquid, the skin grows drier, the pulse more hurried, and eventually the disease appears in its severer form. The febrile character of the affection becomes more marked, the skin of the body and the head are burning hot, the thirst is urgent, and slight delirium comes on. The patients now remain in bed without expressing any desire to get up, and soon they begin to pass their evacuations involuntarily. The dejections are liquid, of a yellowish brown colour, and offensive smell. The abdomen is tympanitic, slightly painful; the skin of the abdomen and of the whole body, with the exception of the feet, is hot; the lips and tongue are dry, but nevertheless, the patients do not ask for drink, though they swallow anything that is given them. They lie for hours together in the same position, altogether unconscious and, to all appearance, in a deep sleep. After three, four, or five days consciousness seems to return, but the little patients seem unable to utter a word or even to protrude their tongue; they lie with their eyes closed, but sleepless; their senses being either weakened or else preternaturally acute. At the end of ten or twelve days there is some slight return of appetite; the tongue, which at no time is thickly coated, becomes moister, and the skin of the lips peels off; but before the end of the third week no critical evacuation takes place, no deposit is thrown down from the urine, no moisture breaks out on the skin. About the beginning of the fourth week, however, perspiration appears about the neck, chest, and abdomen; but after two or three days it ceases and leaves the skin harsher than ever. But now frequent crops of miliary vesicles show themselves, after some hours of restlessness and febrile disturbance; but in the intervals of these attacks the sleep is sound and tranquil. The tongue becomes perfectly clean, very red, broad, and flabby; the thirst ceases, but the patients are troubled by hunger, so importunate that they ask for food every half hour. In about the fifth or sixth week the skin once more becomes perspirable, desquamation of the epidermis takes place, and the hair falls off. The children, though worn to skeletons, may now be regarded as convalescent, but they are still unable to walk; and three, four, or even six months are requisite for their perfect cure, while they are very liable to relapse, from errors in diet or from neglect of the condition of the bowels.

The treatment adopted by Professor Seidlitz is the following: At the commencement of the disease, when premonitory symptoms only exist, he gives very small doses of hydrochloric acid, with ether or Hoffman's anodyne, or else a mixture of *eau de chlore,* in some aromatic infusion. He orders warm baths twice a week, directs the body to be well rubbed with castor oil every day, insists on a very abstemious diet, and gives the patient pure water for drink. The slight diarrhœa which may exist is not to be suppressed, but small doses of castor oil may be given, under the use of which the evacuations gradually become more consistent. If in the course of the disease the evacuations become flocculent and more frequent, an emulsion of olive or poppy oil may be given, and sinapisms may be applied to the hypogastric region or to the feet. If the abdomen become hot and tympanitic, half a drachm of strong mercurial ointment may be rubbed into the hypogastric region twice a day, until the symptoms decline, when recourse may again be had to the acid or chlorine; or, afterwards, to an infusion of the flowers of the *salmiac ferrugineux.* The occurrence of delirium must be met by the application of one or two leeches to the mastoid process, so as to keep up a slight draining of blood for twelve or eighteen hours, and ice must be almost constantly applied to the head till consciousness returns. At the same time, half-grain or grain doses of calomel must be given till they produce green evacuations, when that remedy must be suspended, and the acetate of lead, in quarter-grain doses, may be given three or four times a day, either alone or in combination with musk, in rather large doses, so long as the tympany and liquid stools continue; and, during the same time, mercurial inunctions may be employed every two hours. A small blister may now be applied to the epigastrium, and should, after an hour and a half or two hours, be removed to another part, so as to produce a slight counter-irritation of the skin of the abdomen. After ten or twelve days more the acetate of lead may be discontinued, a camphorated julep may be given instead, and a little Dover's powder may be administered, and a warm bath employed by way of preparing the skin for the future crisis. As soon as the miliary eruption appears all medicine may be discontinued, in the great majority of cases; but the convalescence, usually tardy, is always expedited by removal to a purer air, or by mere change of dwelling.

*Abstracted from Professor Seidlitz's Clinique Médicale, published in the Journal de Médecine, &c., de St. Petersbourg.* Part iii. 1841.

*Insect Origin of Smallpox.* By M. SERRES.

AT the Institut, on the 4th of July, M. Serres mentioned the following fact, seeming to favour the hypothesis of animalcules in smallpox. By covering each pustule with a glass capsule, which is kept for some days in its place, he has seen the process of eruption either go on or languish, or be completely abortive, according as the glass was transparent or more or less opaque. This influence was evidently due to the contact of the air. [Qy.—Should not this be the admission of light?] The experiment, he adds, was not merely curious, for it led to a modification of some of the hygiènic measures adopted in smallpox. Previously patients were generally placed in situations as well aired and lighted as possible; but now one knows that dark situations are far better for this kind of disease, and that this change alone is enough to ensure the most favorable progress of its evolution. The success at La Pitié was never more complete than during one year when all the patients with smallpox had, of necessity, to be put into a low, ill-aired, dark ward, a sort of cellar. The confluent cases there went on as favorably as was possible. At present, in the same hospital, they are moved from the first floor into the *rez-de-chaussée*, and they do well there.

M. Serres took this occasion to mention that he had seen between 1700 and 1800 cases of smallpox in private and in hospital practice, and that he was certain that the number of those affected with smallpox after vaccination was not greater than that of those who had smallpox twice. [For the first of these statements M. Serres may have made it probable, as our old physicians believed, that it is beneficial to exclude the light in smallpox; but there is no evidence that ill ventilation is to be desired.]

*Gazette Médicale. Juillet* 9, 1842.

---

*Case of suddenly-occurring and as suddenly-ceasing Dumbness.*
By Dr. BICKING, of Mühlhausen.

THE patient, a strong lad of fourteen, has, for three successive years, become suddenly dumb in the middle of October and has remained so till the beginning of the following March, when his speech has again suddenly returned. No external cause seems to have acted upon him previous to the occurrence of the dumbness, nor, during its continuance, has he ever shown any sign of general bodily or mental disorder. Medical treatment has been of no avail. [Qy.—May there be imposture here?]

*Oesterreichische Med. Wochenschrift. Mai* 14, 1842.

---

*On a peculiar Granular Appearance of the Buffy Coat of the Blood.*
By M. PIORRY.

IN a recent lecture at the Hospital of la Pitié M. Piorry called the attention of his class to a granular condition of the buffy coat of the blood, which he has observed only in cases in which pus was contained in some organ in considerable quantity, especially in the lungs. In these cases the buffy coat covering the blood contains rounded, grayish granulations, of a darker colour at the centre than at the circumference, varying from the size of a poppy to that of a hempseed. Sometimes five or six of these granulations are contained within the space of a square inch, at other times they are much more numerous. They are semitransparent at their borders, opaque at their centre. They are situated at different depths in the substance of the buffy coat, from which they can be detached with the point of a scalpel, though not easily. The buffy coat does not present any other peculiar appearance, and these granular bodies have never been observed in the clot.

This appearance is far from common: M. Piorry has not met with it above twenty times. In seventeen or eighteen of these twenty cases death took place; and in every instance pus was found in the substance of some organ, especially of the lung, which, in fifteen cases was in a state of purulent infiltration.

Pneumonia with purulent infiltration of the lung existed in the patient whose case gave rise to the above remarks. The granulations are regarded by M. Piorry as collections of purulent matter; and he replies to M. Donné, who denies their resemblance, under the microscope, to the true pus-globule, that the circulation of pus in the blood must alter the characters of the former, and that the results of microscopic investigations are not of value sufficient to overturn facts gathered by clinical observation.

*Gazette des Hôpitaux. Avril* 16, 1842.

